# New Insights into the Phosphorus Acquisition Capacity of Chilean Lowland Quinoa Roots Grown under Low Phosphorus Availability

**DOI:** 10.3390/plants11223043

**Published:** 2022-11-10

**Authors:** Pedro M. de Souza Campos, Sebastián Meier, Arturo Morales, Laura Lavanderos, Javiera Nahuelcura, Antonieta Ruiz, Álvaro López-García, Alex Seguel

**Affiliations:** 1 Instituto de Investigaciones Agropecuarias, INIA Carillanca, km 10 camino Cajón-Vilcún s/n, Temuco P.O. Box 929, Chile; 2 Carrera de Agronomía, Facultad de Ciencias Agropecuarias y Medioambiente, Universidad de La Frontera, P.O. Box 54-D, Temuco 4811230, Chile; 3 Departamento de Ciencias Químicas y Recursos Naturales, Facultad de Ingeniería y Ciencias, Universidad de La Frontera, P.O. Box 54-D, Temuco 4811230, Chile; 4 Instituto Interuniversitario de Investigación del Sistema Tierra en Andalucía (IISTA), Universidad de Jaén, Campus Las Lagunillas, 23071 Jaén, Spain; 5 Departamento de Ciencias Agronómicas y Recursos Naturales, Facultad de Ciencias Agropecuarias y Medioambiente, Universidad de La Frontera, P.O. Box 54-D, Temuco 4811230, Chile

**Keywords:** *Chenopodium quinoa*, phosphorus nutrition, root growth, root exudates, volcanic soil

## Abstract

Reducing phosphate fertilizer inputs while increasing food nutritional quality has been posited as a major challenge to decrease human undernourishment and ensure food security. In this context, quinoa has emerged as a promising crop due to its ability to tolerate different stress conditions and grow in marginal soils with low nutrient content, in addition to the exceptional nutritional quality of its grains. However, there is scarce information about the phosphorus acquisition capacity of quinoa roots. This work aimed to provide new insights into P acquisition and functional root traits, such as root biomass, rhizosphere pH, carboxylate exudation, and acid phosphatase activity of thirty quinoa genotypes grown under P limiting conditions (7 mg P kg^−1^). Significant genotypic variation was observed among genotypes, with average P accumulation ranging from 1.2 to 11.8 mg. The shoot biomass production varied more than 14 times among genotypes and was correlated with the P accumulation on shoots (r = 0.91). Despite showing high variability in root traits, only root biomass production highly correlated with P acquisition (r = 0.77), suggesting that root growth/morphology rather than the measured biochemical activity possesses a critical role in the P nutrition of quinoa.

## 1. Introduction

One of the most important challenges for modern agriculture is to reduce undernourishment and ensure global food security without expanding arable land—in order to preserve natural ecosystems and, at the same time, reduce agricultural inputs, especially the non-renewable ones such as phosphate fertilizers [1]. Unlike nitrogen, which can be fixed from the atmosphere through biological and industrial processes—a virtually infinite reservoir, the dependence on phosphate fertilizers is unsustainable, as it is manufactured from phosphate rocks, which have finite stocks [2]. In addition, phosphate rock reserves are unevenly distributed worldwide, being concentrated in a few countries [3]. Although estimates of the global phosphorus (P) reserves vary widely, recent reassessments suggest that they may be depleted even within this century [2,4], highlighting the need for more efficient use of this resource.

Among the widely suggested approaches to accomplishing future food security, improving the nutritional value of food has been recognized for its global coverage and long-term cost-effectiveness [5]. In this context, while sustainable biofortification of traditional crops remains a challenge, the use of “forgotten” highly nutritious crops which are adapted to grow in marginal soils emerges as an opportunity to produce quality food [5,6,7].

Quinoa (*Chenopodium quinoa* Willd.) has gained interest as a promising crop to fill this gap due to its capacity to tolerate a wide range of stress conditions, including its ability to grow in soils with low nutrient content and under conditions of drought, plus the exceptional nutritional quality of its grains [6,8]. Although some agronomical practices such as sowing date, plant density, and control of weeds and diseases, among others, have been studied in recent years [9,10,11,12], there is not enough information about nutrient management in this crop, especially regarding low-mobile plant nutrients such as phosphorus. In detail, there is a lack of information about the P-acquisition capacity of quinoa roots and their genotypic variability to promote sustainable use of P resources for this species.

Roots perform essential functions throughout plant development, such as providing anchorage into the soil, resource storage, synthesis of essential metabolites, supporting soil microbial communities, water uptake, and acquisition of nutrients—especially for those with low mobility in soils such as P [13]. Accordingly, selecting for root traits that maximize nutrient and water uptake efficiency has been indicated as a key target for a “Second Green Revolution” due to their potential to achieve more sustainable agricultural production [14,15,16].

A great variety of regulatory mechanisms are displayed by roots to enhance resource acquisition under non-optimal conditions [17]. In the case of P, the main root adaptations for increasing its uptake have been intensively reviewed [18,19,20] and include: (i) greater carbon allocation to root biomass production, modifications in root system architecture, and morphological adaptations [21,22]; (ii) “mining” strategies, through actively changing soil pH, the activity of nutrient releasing enzymes, such as phosphatases, and exudation of carboxylates [23]; and (iii) association with soil microorganisms, especially mycorrhizal fungi [24]. However, considering that quinoa is a non-mycorrhizal plant (or with a very low dependency on this symbiosis) [25], the other strategies should have a relative greater importance in increasing the acquisition of nutrients with low mobility in soil, especially P, which allows quinoa plants to grow in marginal soils that warrant more studies.

Finally, the complex of southern Chilean lowland quinoa ecotypes represents a unique genetic reservoir of plants adapted from the Mediterranean to temperate climates [9,26], which, due to their ancestral origin adapted to volcanic high P-fixing soils, would allow the selection of efficient genotypes able to produce high-quality grains with fewer P inputs. This study aimed to provide one of the first insights into the P-acquisition strategies developed by quinoa roots grown under low P availability and their phenotypic variability on a set of Chilean lowland quinoa accessions.

## 2. Results and Discussion

**Figure 1:**
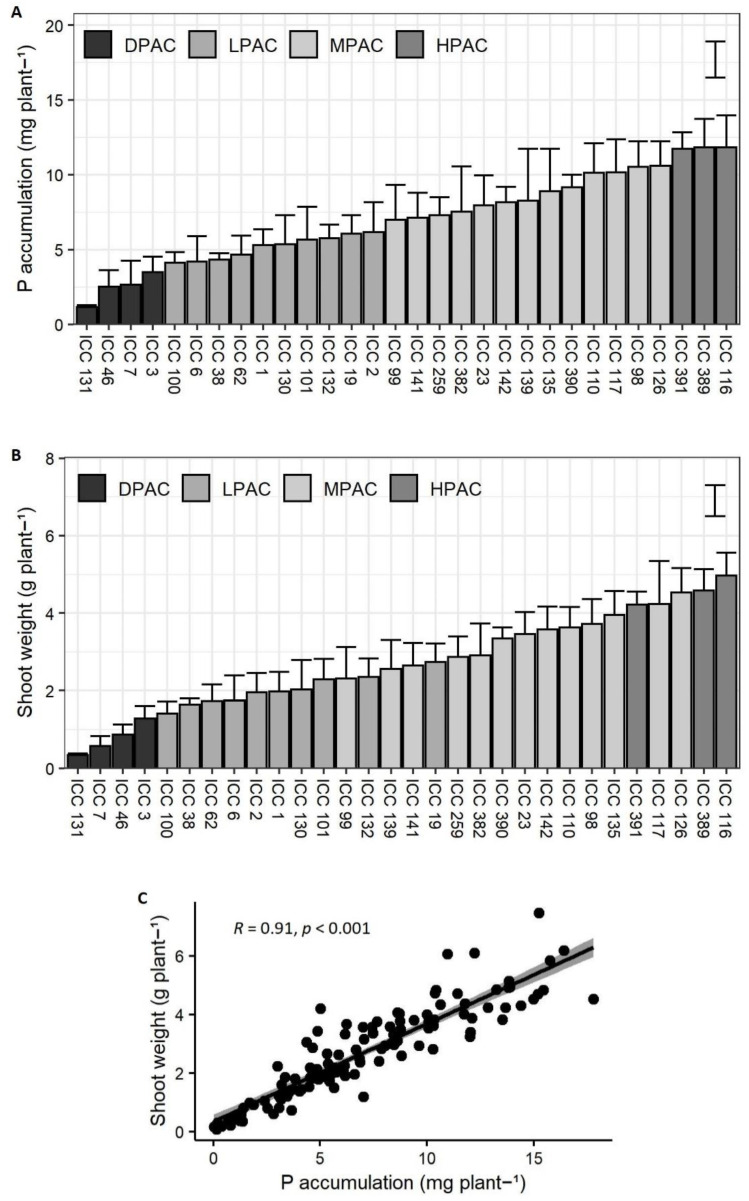
Growth and P accumulation of thirty Chilean quinoa accessions after 13 weeks of plants growing on a volcanic soil with low available P. Graphs representing phosphorus accumulation in biomass per plant (**A**), shoot biomass production per plant (**B**), and Pearson correlation analysis among both variables (**C**). Error bars result from the mean of four biological replicates (±SE). Bars in (**A**,**B**) represent least significant difference (LSD) at *p* = 0.05 among genotypes. Abbreviations: high P acquisition capacity (HPAC), medium P acquisition capacity (MPAC), low P acquisition capacity (LPAC), and deficient P acquisition capacity (DPAC).

A significant and considerable variation among genotypes was found for P accumulation in biomass (*p* < 0.001; F-value = 2.9; Figure 1A). After determining the mean and standard deviation of the group of accessions for P accumulation, the quinoa accessions were classified into four categories based on a modified categorization from Meier et al. (2021) [27], as follows: high P acquisition capacity (HPAC), medium P acquisition capacity (MPAC), low P acquisition capacity (LPAC), and deficient P acquisition capacity (DPAC) (Figure 1A). Accordingly, a 4.6-fold difference was found for average P accumulation between genotypes with higher P acquisition (ICC 116, ICC 389, and ICC 391) and those with deficient P acquisition (ICC 131, ICC 46, ICC 7, and ICC 3).

Average shoot biomass production per accession was in the range of 0.34–4.9 g of dry matter (Figure 1B). Shoot growth was significantly different among the P acquisition classes (*p* < 0.001; F-value = 4.8) and directly correlated with P accumulation (r = 0.91, *p* < 0.001; Figure 1C). Accordingly, genotypes with DPAC showed the lowest shoot biomass production, while ICC 116 and ICC 389, classified as HPAC, achieved the highest values. Together, these results suggest that a greater P acquisition capacity is an essential trait for sustaining quinoa growth under low available P levels. On the other hand, ICC 126 and ICC 117, which were classified as MPAC accessions, showed greater shoot biomass production than ICC 391, an HPAC genotype (Figure 1B). The former could be related to a higher internal P use efficiency of these genotypes, which may require less P for producing biomass compared to ICC 391 [28].

**Figure 2:**
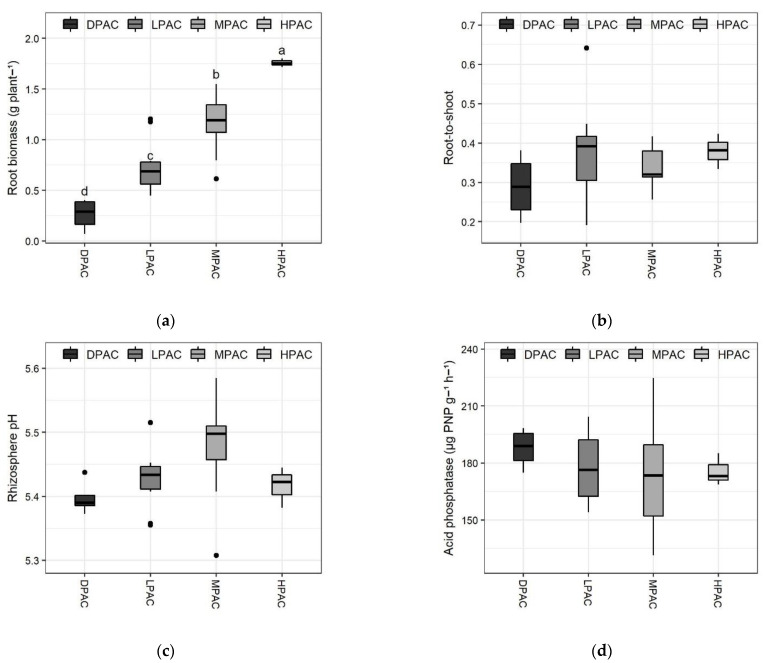

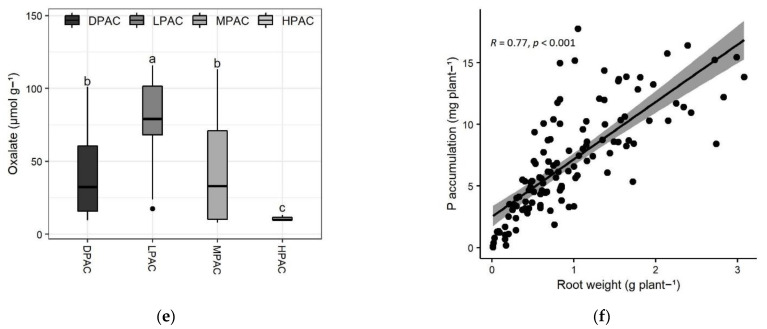
Main root traits of the Chilean quinoa accessions belonging to the different P acquisition classes after 13 weeks of plants growing on a volcanic soil with low available P. Boxplots representing root biomass per plant (**a**), root-to-shoot ratio (**b**), pH in the rhizosphere (1:2.5 soil:water) (**c**), acid phosphatase activity in the rhizosphere (**d**), oxalate concentration in the rhizosphere (**e**), and Pearson correlation analysis between root weight and P accumulation (**f**). Means with different letters are significantly different according to the Tukey HSD test (*p* < 0.05). Abbreviations: high P acquisition capacity (HPAC), medium P acquisition capacity (MPAC), low P acquisition capacity (LPAC), and deficient P acquisition capacity (DPAC).

Root biomass production varied greatly among genotypes and differed significantly between the distinct P acquisition classes (*p* < 0.001; F-value = 2.6; Figure 2a). The three genotypes classified as HPAC showed the highest root biomass production (>1.7 g per plant), whereas the four genotypes belonging to the DPAC class presented the lowest values (<0.4 g per plant) (Figure 2a). Root biomass showed the highest correlation with P acquisition among the analyzed root traits in this study (r = 0.77; *p* < 0.001; Figure 2f). Root-to-shoot ratio showed less variability compared to their individual components (i.e., root or shoot biomass production), ranging from 0.2 to 0.45, except for ICC 6, which presented a root-to-shoot ratio of 0.64 (Figure 2b). No significant differences were found in the root-to-shoot ratio among the different P acquisition classes.

All quinoa genotypes acidified the rhizosphere soil, except ICC 135, whose rhizosphere pH remained at 5.58 (Figure 2c). The pH on the rhizosphere ranged from 5.31 to 5.58 among accessions; that is, quinoa plants reduced rhizosphere soil pH up to 0.27 units. A similar pH reduction has been observed for other plant species, such as lupins, maize, rice, and alfalfa [29,30,31]. Plants-induced changes in soil pH are often attributed to root respiration, carboxylate exudation, and/or cationic-anionic equilibrium in soil solution due to preferential nutrient uptake of a given ionic form [30,31,32]. Accordingly, soil pH is a key factor determining P availability for plants; however, species-specific optimum pH for root P uptake does not always match with maximum P availability dependent on soil pH [18]. No significant differences were observed between the P acquisition capacity classes.

A 1.7-fold difference in acid phosphatase (Pase) activity in the rhizosphere was observed among the 30 genotypes, with average values per accession ranging from 131.2 to 224.8 µg PNP g^−1^ h^−1^ of rhizosphere soil (Figure 2d). Organic P forms can comprise up to 80% of total P in some soils [33]; thus, it has been suggested that greater P mineralization through Pases could represent an important approach for reducing P fertilizer inputs [34]. However, greater phosphatase activity does not always imply improved plant P acquisition, as their effectiveness depends on many factors, mainly soil mineralogy [35]. Here, Pase activity in the rhizosphere of quinoa plants did not significantly differ between the P-acquisition capacity classes.

Oxalate was the predominant carboxylate found in the rhizosphere of the quinoa accessions, with a 14.4-fold difference between genotypes (Figure 2e). Oxalate concentration was in the range of 8–115 µmol g^−1^ of rhizosphere soil. Six accessions (ICC 100, ICC 98, ICC 38, ICC 259, ICC 6, and ICC 7) had the highest levels of oxalate concentration (>100 µmol g^−1^), whereas four accessions (ICC 142, ICC 390, ICC 46, and ICC 391) had the lowest values (<10 µmol g^−1^). High carboxylate exudation is a well-known root adaptation for mobilizing sparingly available P forms, especially on severely impoverished and/or high P-fixing soils [36]. Notwithstanding, in our study, oxalate concentration in the rhizosphere soil negatively correlated with P acquisition (r = −0.23; *p* < 0.05; not shown) and was greater on LPAC genotypes. This negative relationship was also observed in other studies [37,38,39] and could be related, among other factors, to a stronger organic P mobilization than inorganic readily absorbable P when soil organic matter is high, as in our case [40].

**Figure 3:**
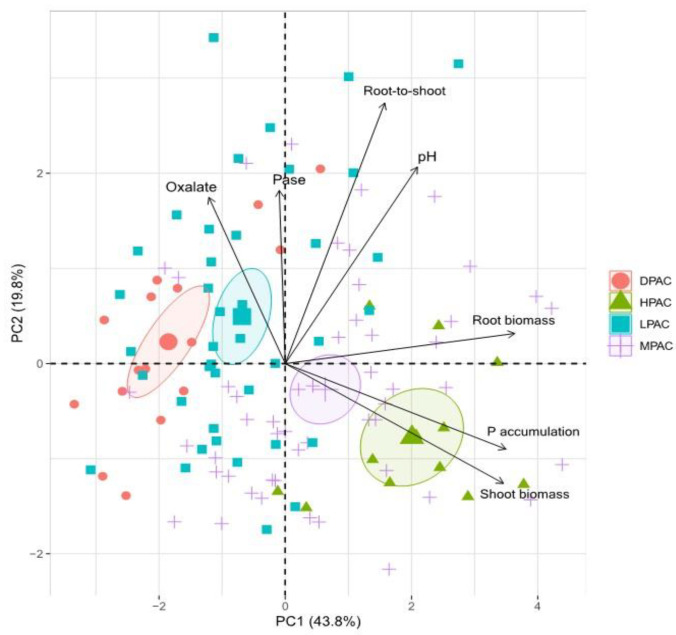
Principal component analysis of seven traits of 30 Chilean quinoa genotypes grown for 13 weeks in a volcanic soil with low available P. Biplot vectors are trait factor loadings, whereas the position of individual plants is shown. Abbreviations: high P acquisition capacity (HPAC), medium P acquisition capacity (MPAC), low P acquisition capacity (LPAC), and deficient P acquisition capacity (DPAC).

The Principal Component Analysis (PCA) based on seven traits of 30 quinoa genotypes explained 63.6% of the total variance in the first two components (Figure 3). The first component represented 43.8% of the variability and was mainly affected by root biomass, shoot P accumulation, and shoot biomass. The second component represented 19.8% of the variance and accounted primarily for root-to-shoot ratio, rhizosphere pH, phosphatase activity (Pase), and oxalate concentration. PCA visualization tended to generate homogenous groups between individuals and suggested a continuous divergence among the different P acquisition capacity accessions (Figure 3).

This study showed that Chilean lowland quinoa accessions possess a high genotypic variability regarding P acquisition capacity under restricted available-P conditions. The former could be related to differences in root morphological/architectural traits associated with root biomass production rather than their biochemical activity. However, it is important to point out that a single trait may not invariably explain higher P accumulation, as the diversity of P forms present in soils, different edaphoclimatic site-specific conditions, and the trade-offs on carbon cost in the root traits spectrum must be properly accounted. Further studies have to be carried out to deepen understanding of the mechanisms related to the P acquisition of quinoa plants as well as the responsiveness of contrasting genotypes to increasing soil P levels and their genetic background.

## 3. Materials and Methods

Thirty Chilean lowland quinoa accessions conserved in the Germplasm Bank of the Regional Research Center Carillanca of the Institute of Agricultural Research (INIA) (Vilcún, Chile) were used in this study. These genotypes were collected in Chile between −35° S and −40° S, from O’Higgins to Los Rios regions, corresponding to Mediterranean and Temperate climate zones. More details regarding the quinoa accessions are available in Supplementary Table S1.

Plastic pots (20 cm × 20 cm × 25 cm; width × length × depth) were filled with 5 kg of air-dried agricultural volcanic soil (Barros Arana Series, medial, mesic, Typic Hapludand, Ciren 2002) with 7 mg kg^−1^ of available P (Olsen-P), which was previously ground to pass through a 5mm sieve and thoroughly homogenized. Soil chemical characterization was performed according to Sadzawka et al. (2006) [41] (Table 1). Nitrogen and potassium were provided on an equivalent dose of 200 kg ha^−1^ of N (split into two applications) and 140 kg ha^−1^ of K_2_O as commercial fertilizers (calcium ammonium nitrate and polysulphate, as sources of N and K, respectively). Each pot was planted with six seeds at 15 mm depth, with four replicates per accession (*n* = 120). Seedlings were thinned to one plant per pot 12 d after sowing. Plants were grown under glasshouse conditions for 13 weeks from September to December 2021 at the Regional Research Center Carillanca of the INIA, Vilcún, Chile (38°41′ S 72°25′ W), and were watered every other week.

At harvest, shoots were separated from roots, and loose soil was removed by gently shaking the root system. The soil firmly attached to roots, hereafter defined as rhizosphere soil, was carefully collected with forceps and stored at 4 °C for approximately two weeks until further analysis. Immediately after harvest, roots and shoots were cleaned with distilled water, oven-dried at 70 °C for 72 h, and weighed. Then, shoots were ground, ashed, digested in an HNO_3_:HClO_4_ solution, and P concentration was determined by spectrophotometry using the vanado-molybdate method [45]. P accumulation per plant was calculated by multiplying P concentration and shoot dry weight of each sample.

Rhizosphere soil pH was determined on 10 g of dried soil (1:2.5 soil:water). Acid phosphatase activity in the rhizosphere was determined by incubating 1 g of rhizosphere soil in the Modified Universal Buffer (pH 6.5) with 25 mM of p-nitrophenyl phosphate (PNP) as substrate at 35 °C for 1 h [46]. The identification and quantification of carboxylates on rhizosphere soil were performed as described in de Souza Campos et al. (2021) [47]. Briefly, 2 g of rhizosphere soil were placed in a 50 mL tube containing 10 mL of CaCl_2_ (0.2 mM), shaken, centrifuged, filtered (0.22 µm pore), and analyzed by high-performance liquid chromatography with diode array detection (HPLC DAD) equipped with an LC-20AT quaternary pump, a DGU-20A5R degassing unit, a CTO-20A oven, a SIL-20a autosampler, and an array of UV visible detector diode (SPD M20A) (Shimadzu, Tokyo, Japan). Control and data collection were performed using Lab Solutions software (Shimadzu, Duisburg, Germany). The chromatographic separation method was performed based on what was reported by Parada et al. [48].

Genotypes were classified into four categories based on their P acquisition capacity using a modified classification method described in Meier et al. (2021) [27]. The genotypes were assigned with HPAC if their mean P accumulation was higher than the mean plus standard deviation of the total population of genotypes evaluated (>μ + SD), MPAC if their mean value was between μ and μ + SD, with LPAC if their mean value was between μ and μ − SD, and with DPAC if their mean P accumulation was lower than the mean less the standard deviation of the entire population (<μ − SD).

A one-way ANOVA was used to evaluate significant differences in P accumulation and shoot biomass production among accessions. Then, another one-way ANOVA was performed to evaluate significant differences in root traits across P-acquisition capacity classes, followed by the Tukey HSD test when suitable. Pearson correlations and Principal Component Analysis were performed between the main variables under study. All analyses were carried out using RStudio software (RStudio, Inc., version 2022.07.1, Boston, MA, USA) using factoextra, factominer, corrr, ggpubr, car, and agricolae packages.

## Figures and Tables

**Table 1:** Main chemical properties of the soil used in the experiment.

Parameter	Value
N (mg kg^−1^) ^a^	40.7
P (mg kg^−1^) ^b^	7.0
K (mg kg^−1^) ^c^	176.1
pH ^d^	5.58
Organic matter (%) ^e^	21.0

^a^ Total Nitrogen by Kjeldahl method [42], ^b^ Available P by Olsen method [43], ^c^ Extracted with ammonium acetate (1 M) and determined by atomic absorption spectroscopy [41], ^d^ Measured in H2O, ^e^ Determined by soil oxidation according to Walkey–Black method [44].

## Data Availability

Data can be available upon personal request.

## Supplementary Materials

The following supporting information can be downloaded at: https://www.mdpi.com/article/10.3390/plants11223043/s1, Table S1: Accession codes available on GRIN-Global database.
