# Supplementary material for: New Insights into the Phosphorus Acquisition Capacity of Chilean Lowland Quinoa Roots Grown under Low Phosphorus Availability
Source: Plants (Basel). 2022 Nov 10;11(22):3043. doi: 10.3390/plants11223043 (PMC9695380; doi:10.3390/plants11223043)
Supplement: Supplementary file 1 [file plants-11-03043-s001.zip › plants-1982800-supplementary.pdf]

plants

Supplementary Material

Table S1. Accession numbers and their respective code on GRIN-Global database (www.grin-global.org, accessed on 4 October 2022).

Accession	Observation	GRIN-Global Code
ICC 1	INIA Quinoa breeding program	Not available
ICC 2	INIA Quinoa breeding program	Not available
ICC 3	INIA Quinoa breeding program	Not available
ICC 6	INIA Quinoa breeding program	Not available
ICC 7	INIA Quinoa breeding program	Not available
ICC 19	INIA Quinoa breeding program	Not available
ICC 23	INIA Seed bank	CQU 397
ICC 38	INIA Quinoa breeding program	Not available
ICC 46	INIA Quinoa breeding program	Not available
ICC 62	INIA Quinoa breeding program	Not available
ICC 98	INIA Gene bank	CQU 98
ICC 99	INIA Quinoa breeding program	Not available
ICC 100	INIA Quinoa breeding program	Not available
ICC 101	INIA Gene bank	CQU 101
ICC 110	INIA Gene bank	CQU 110
ICC 116	INIA Gene bank	CQU 116
ICC 117	INIA Gene bank	CQU 117
ICC 126	INIA Gene bank	CQU 126
ICC 130	INIA Gene bank	CQU 130
ICC 131	INIA Gene bank	CQU 131
ICC 132	INIA Gene bank	CQU 132
ICC 135	INIA Gene bank	CQU 135
ICC 139	INIA Gene bank	CQU 139
ICC 141	INIA Gene bank	CQU 141
ICC 142	INIA Gene bank	CQU 142
ICC 259	INIA Quinoa breeding program	Not available
ICC 382	INIA Gene bank	CQU 382
ICC 389	INIA Gene bank	CQU 389
ICC 390	INIA Gene bank	CQU 390
ICC 391	INIA Gene bank	CQU 391
